# New Ceramic Multi-Unit Dental Abutments with an Antimicrobial Glassy Coating

**DOI:** 10.3390/ma15155422

**Published:** 2022-08-06

**Authors:** Roberto López-Píriz, Lidia Goyos-Ball, Belén Cabal, Susana Martínez, José S. Moya, José F. Bartolomé, Ramón Torrecillas

**Affiliations:** 1 Instituto de Cirugía Oral Avanzada-ICOA, Calle de Fray Luis de León, 14, 28012 Madrid, Spain; 2 Nanoker Research, Pol. Ind. Olloniego, Parcela 22A, Nave 5, 33660 Oviedo, Spain; 3 Nanomaterials and Nanotechnology Research Centre (CINN), Consejo Superior de Investigaciones Científicas (CSIC), Universidad de Oviedo (UO), Principado de Asturias (PA), Avenida de la Vega 4-6, 33940 El Entrego, Spain; 4 Instituto de Ciencia de Materiales de Madrid (ICMM), Consejo Superior de Investigaciones Científicas (CSIC), 28049 Madrid, Spain

**Keywords:** coating, dental implant, peri-implantitis, glass, biocide, Ce–TZP/Al_2_O_3_ ceramic composite

## Abstract

The choice of suitable materials and new designs in oral implantology and the subsequent enhancement of the characteristics of the dental implant developed is an important research topic with wide scope. The present work aims to develop a new multifunctional zirconia–ceria/alumina (Ce–TZP/Al_2_O_3_) composite with an antimicrobial glass-based coating to be used in multi-unit abutments compatible with commercially available Ti implants for peri-implantitis prevention. An airbrush spraying technique was effectively applied to coat the sintered ceramic composite starting from a glass powder suspension. This deposition technique was appropriate for obtaining continuous antimicrobial glass-based coatings with homogenous thickness (~35 µm) on ceramic dental implant components. The dental implant systems with the antimicrobial glassy coating were subjected to a mechanical integrity test following ISO 14801 to determine their long-term stability. The tested implant-coating structure seems to be stable under in vitro conditions with ultimate applied forces exceeding the maximum physiological occlusal loading force. This paper also presents a pilot clinical case report that shows peri-implant tissue around the mechanically stable glass coating with no signs of inflammation 1 year after implant insertion. This result is a preliminary probe of the durability and biological tolerance of the glassy material by the gingiva, as well as the antimicrobial effect on the peri-implant microbiota displayed by the coating.

## 1. Introduction

To date, the research found in the literature on oral implantology has focused on the biological and mechanical performance of the biomaterials developed [1,2]. Nowadays, the challenge is to design dental implants that have a multifunctional character, combining properties such as antimicrobial activity, biocompatibility, aesthetics, prevention of biofilm formation, osseointegration, non-toxicity, etc. Despite the long history and solid establishment of titanium and titanium alloy dental implants in the market, the search for more aesthetic and biologically less controversial alternatives to metals continues. Yttria-stabilized zirconia (YTZ) ceramics have been postulated as an alternative that meets these requirements; however, their intrinsic mechanical properties and the low-temperature degradation issue are, at present, important limitations in the design of long-term and stable YTZ ceramic implants [3]. Various authors have pointed out the need for technical advances in ceramics to enable the application of modern protocols in terms of prosthetic versatility and tissue preservation, especially in the aesthetic sector [4,5,6]. In the current study, we used a new ceramic implant component as an alternative to the commercially available ones, based on a composite zirconia–ceria/alumina ceramic material that has shown perfect biocompatibility, a high rate of osseous integration and soft tissue attachment [7], superior mechanical properties (i.e., K_IC_ > 10 MPa∙m^1/2^) to biomedical grade yttria-stabilized zirconia (Y–TZP) [8] and insensitivity to low-temperature degradation [9,10].

The potential for new materials in dental implantology is of great significance, particularly with respect to long-term prosthetic implant therapy. The main problem in a large number of implants is marginal crestal bone loss. Peri-implantitis has a prevalence of up to 43% of implants and 56% of subjects. This disease originates as a consequence of a dysbiosis in the peri-implant groove in a similar way to periodontitis [11]. At present, it is very well documented that one of the most prevalent causes of peri-implantitis is bacterial leakage at the implant–abutment connection. Microgaps at this interface and the infiltration of microbiota between these two components are important factors in chronic inflammation and marginal bone resorption [12,13]. Prevention of bacterial leakage improves the bone stability and reduces the inflammation processes around implant necks. Furthermore, the presence of Ti products around dental implants may contribute to peri-implantitis [14]. In the oral cavity, fluctuations in temperature, pH, oxygen, bacteria and food decomposition continuously attack the implant surface. Furthermore, the interactions between dental implants and prosthetic superstructures (produced by the differences in the electric potential of the materials) can create crevices, pitting and galvanic corrosion, leading to the subsequent dissolution of the pure metal and alloy components. Corrosion could also occur under mechanical loading, through a phenomenon where the implant surface and bone can suffer a small amplitude, oscillatory, relative motion (fretting) in which chemical reactions prevail. Ti ions can change the microbiological composition of the bacterial biofilms produced on Ti surfaces [15].

Apart from the already known collateral problems (metabolic syndrome (MetS), cardiovascular disease (CVD), Alzheimer’s disease, etc.) of peri-implant disease [16,17,18], very recently, the relationship between this disease and greater severity of COVID-19 has been reported [19]. Marouf et al. [19] reported that patients with periodontitis are nine times more likely to die from COVID-19 than those without it. Consequently, the development of new materials is interesting for a preventive approach and the treatment of diseases, as well as for avoiding the arbitrary prescription of antibiotics. For example, dentistry accounts for 13% of all antibiotic prescriptions in the USA [20]. To date, several antibacterial coatings have been developed for managing peri-implantitis [21,22,23,24]. In an attempt to prevent or treat bacterial colonization of dental implant components, metallic nanoparticles have been investigated as a coating material [25,26,27,28,29]. However, the main disadvantage of this approach is the latent toxicity and accumulation of metals over time [30]. The unanswered questions about the cytotoxicity of these compounds upon long-term exposure have triggered great efforts to identify new possibilities to fight peri-implantitis.

In this context, the search for multifunctional ceramic implants with improved long-term performance, aesthetic appearance, osseointegration and non-cytotoxicity, as well as effective protection against peri-implantitis, is becoming ever more urgent. In view of the biological issues related to the peri-implantitis issue, non-cytotoxic antimicrobial glasses and glass ceramics have attracted special attention as coatings for dental applications. Esteban-Tejeda et al. [31] successfully tested antimicrobial coatings made of a soda–lime glass belonging to the SiO_2_-Na_2_O-Al_2_O_3_-CaO-B_2_O_3_ system. The antibacterial activity of this glass, free of P_2_O_5_, effectively diminished the growth (logarithm of reduction >3) of bacteria [32]. López-Píriz et al. [27] evaluated the efficacy of this antimicrobial glassy coating in preventing peri-implant bone loss, intrasulcular bacterial growth and biofilm formation in a ligature-induced peri-implantitis model. Furthermore, Rius-Rocabert et al. [33] demonstrated that the antimicrobial glass shows strong antiviral properties (a reduction in viral infectivity higher than 99.9% after 1 h of contact) against model viruses such as the enveloped viruses vesicular stomatitis virus, influenza virus, pandemic virus SARS-CoV-2 or herpes simplex virus type I (HSV-1) and the non-enveloped adenovirus. Additionally, the mechanical performance, bioactivity, biocompatibility and non-cytotoxicity of the products released from these glassy materials were previously confirmed [34,35,36,37,38]. The results obtained in these investigations proved the viability of producing new glassy coatings with anti-infective properties. Based on these results, in the present investigation, a glass belonging to the SiO_2_-Na_2_O-Al_2_O_3_-CaO-B_2_O_3_ system was selected and applied as a protective vitreous coating on zirconia–ceria/alumina new-generation ceramic implant components. This glassy coating with proven antimicrobial character was applied on the surfaces of ceramic components that were to be in direct contact with the gum, with the intention of protecting the implant from the future action of the anaerobic bacteria that cause peri-implant disease.

In the present study, a complete evaluation of this new family of coated ceramic implant components is performed. Given the extraordinary importance that peri-implant disease has nowadays as a major public health issue, special emphasis has been placed on the development of a glassy coating to protect the patient from the action of anaerobic bacteria that induce the appearance of peri-implant disease, together with the assessment of its long-term integrity and mechanical stability. Finally, coated implants were placed in a patient with periodontal problems and the evolution followed over a period of 12 months.

## 2. Materials and Methods

### 2.1. Dental Implant Components 

**Figure 1:**
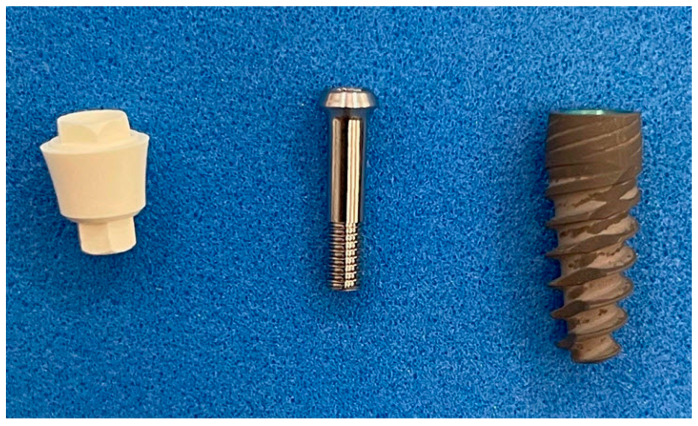
Zirconia–ceria/alumina composite abutment (left), Ti 6Al-4V ELI screw (center) and JD EVOLUTION PLUS+ commercial titanium implant (right).

Ceramic abutments to fit JD EVOLUTION PLUS+ commercial titanium implants (Figure 1) were manufactured using a zirconia–ceria/alumina composite ceramic material (Nanoker Research S.L., Oviedo, Spain). During production, the material undergoes uniaxial and cold isostatic pressing before it is subjected to a complex and very sensitive debinding/pre-sintering cycle. The material is machined in a pre-sintered state. After machining, the pieces are sintered, and once sintered they are subjected to all the established quality control verifications.

In addition, Ti- 6Al-4V ELI screws were designed and manufactured to fit and fix the ceramic abutments to the JD EVOLUTION PLUS+ implants. This commercially available titanium implant was selected because of the adaptability of its design to ceramic processing requirements and possibilities. In addition, it was thought that coated ceramic abutments could become of interest for titanium implant manufacturers in the future, since they can be applied on implants that are still to be placed in the mouths of patients or on those that have already been placed (millions of units).

### 2.2. Preparation of the Coatings

**Figure 2:**
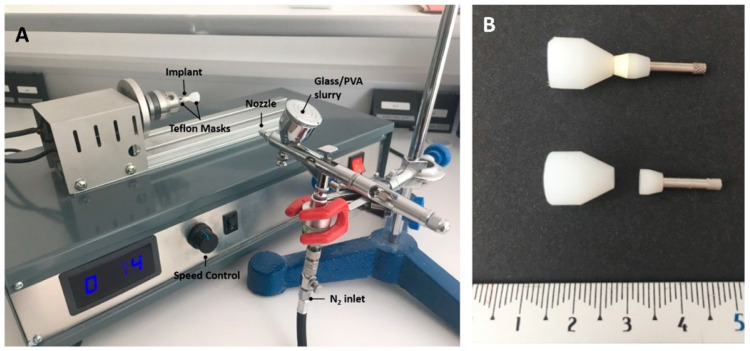
(**A**) Image of the device used for the coatings. (**B**) Photograph of the masks, with (above) and without (below) the abutment.

The material used for coating preparation was an antimicrobial soda–lime glass with a thermal expansion coefficient of 14.2·10^−6^ K^−1^ [28], with the following composition (wt.%): 41.6 SiO_2_, 20.0 Na_2_O, 19.5 CaO, 10.1 Al_2_O_3_, 6.4 B_2_O_3_, 0.21 MgO and 0.61 K_2_O. This glass was synthesized via a melting route (Nanoker Research S.L., Oviedo, Spain). The glass frit was milled and sieved to obtain a particle size of <50 µm (d_50_: 13 μm; d_90_: 36 μm) with a faceted and irregular morphology. A slurry was produced from the glass, with 80% hydrolyzed 0.39 wt.% poly (vinyl alcohol) (PVA) (Merck KGaA, Darmstadt, Germany) used as the binder and with a solids content of 48 wt.%. The glass was added after PVA hydrolysis. The PVA solution was prepared in water at 80 °C. The slurry was prepared under continuous mechanical stirring to ensure homogeneity. The layer-wise slurry deposition on the ceramic abutment was performed using an airbrush spray gun (Iwata Custom Micron, Racing Colors, Barcelona, Spain). A special device was designed for the controlled rotation of the abutment while projecting the glass onto it (see Figure 2A). The rotational speed was kept constant (74 rpm). The use of tailor-made Teflon masks facilitated coated abutment manipulation and ensured the reproducibility of the coated area on all specimens (Figure 2B).

Some important processing variables such as nozzle diameter, nozzle-to-substrate distance, speed of the spraying movement and air pressure were kept constant. The quantity of the spraying mixture was kept constant by controlling the optimal total spraying time (1 min) and the spray pressure, selected on the basis of preliminary experiments. 

Single green layers were dried overnight under ambient conditions and subsequently thermally treated in an electrical furnace at 1200 °C for 5 min (heating rate 50 °C min^−1^). The cooling rate was rapid enough to avoid crystallization, as well as any cracking or chipping phenomena. Coated and non-coated abutments were weighed before and after the thermal treatment.

The coated parts were cleaned and subjected to autoclave sterilization (at 121 °C and 1 atm for 20 min in a J.P. Selecta autoclave (J.P. Selecta, Barcelona, Spain).

### 2.3. Characterization of the Coatings

The coatings were characterized via X-ray diffraction (XRD) using a Bruker D8 Discover diffractometer (Bruker, Karlsruhe, Germany). Diffractograms were performed in a step scan mode from 4° to 70°, with a step size of 0.05° and a step time of 0.5 s, using CuKα radiation and working at 40 kV with an intensity of 30 mA.

The glass-coated abutments were embedded in a thermoplastic acrylic mounting material (TransOptic) (Buehler, Lake Bluff, IL, USA) and polished using #220 and #1200 MD-Piano diamond grinding discs and diamond polishing sprays (Struers, Ballerup, Denmark) of 9, 3 and 1 µm. The obtained cross sections were analyzed using field emission scanning electron microscopy (FESEM) (FEI Quanta FEG 650, ThermoFisher Scientific, Waltham, MA, USA).

### 2.4. Cytotoxicity Testing

The potential cytotoxic effects of the coated and uncoated abutments were evaluated following the guidelines of ISO 10993 [39]. Cell viability was evaluated using a neutral red uptake (NRU) staining assay, which allows in vitro quantification of xenobiotic-induced cytotoxicity. Osteoblast-like cells were selected as mammalian cells for this experiment. The SaOS-2 cell line (Homo sapiens bone osteosarcoma, 11-years old Caucasian female; morphology: epithelial; culture properties: adherent) was kindly supplied by the Unit of Biotechnology and Biomedicine, Scientific Technical Services at the University of Oviedo (SCT-UNIOVI), Oviedo, Spain. Three samples of the coated and uncoated abutments were incubated for 24 h at 37 °C in order to obtain complete extracts. These eluents were used as the cell culture medium for osteoblasts grown for 24 h (in triplicate). After adding the desorption reagent, absorbance at a wavelength of 540 nm, which is directly proportional to the number of living cells in the culture, was measured using a BIO-RAD Model 680 microplate reader (Bio Rad Laboratories, Madrid, Spain). All assays were performed in triplicate. Three controls were used in this experiment: a positive control with wells containing cells and culture medium with 2 vol% Triton X-100 (Sigma-Aldrich (Merck KGaA, Darmstadt, Germany)), a negative control with wells containing cells and culture medium and a blank control with empty wells containing culture medium.

### 2.5. Mechanical Testing

**Figure 3:**
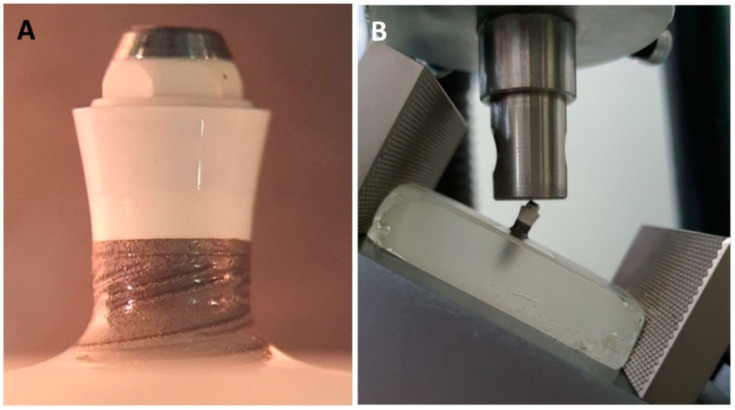
(**A**) Embedded specimens simulating vertical bone resorption. (**B**) Test setup for the fatigue tests.

The mechanical durability of the coated implant systems was measured through cyclic fatigue loading tests, following ISO 14801 [40]. An epoxy resin (Epoxycure resin, Buhler, Lake Bluff, MN, USA) with an elastic modulus of approximately 4 GPa (similar to the human mandibular [41]) was used as an embedding material. Specimens were embedded with a 3 mm vertical distance from the most coronal bone-to-implant border to the top of the holder (Figure 3A), simulating vertical bone resorption following the method previously described [42,43,44,45,46,47]. The implants were placed at an angle of 90 degrees to the horizontal plane in the center of a silicon mold (ATM-M^®®^, Mammelzen, Germany) with dimensions of length = 55 mm, width = 30 mm and height = 22 mm, containing the resin. The use of a prefabricated external fixation device allowed precisely perpendicular embedding of all implants.

Specimens were tested using an electromagnetic testing machine (Shimadzu EMT series^®^ EMT-1KN-30, Shimadzu^®^, Kyoto, Japan) under load control. A unidirectional and tilted (30°) pulsating load was applied on the surface of the implant in a dry environment with a sinusoidal waveform (Figure 3B). The stress ratio (R), i.e., the minimum-to-maximum loading ratio, was equal to 10, and the loading frequency was fixed at 15 Hz. The cyclic forces selected for the test were between −40 and −400 N (140 N.cm), simulating forces generated in the oral cavity [48,49]. The fatigue life test was interrupted at Nf = 10 × 10^6^ cycles, which would be equivalent to a masticatory function of approximately 20 years of service [50]. Five specimens were evaluated. The surfaces of the coatings were examined using optical microscopy and scanning electron microscopy (SEM), before and after the mechanical tests.

### 2.6. Preliminary Clinical Study

The clinical study was performed at a single center (Instituto de Cirugía Oral Avanzada-ICOA, Madrid, Spain). The patient signed the informed consent form approved by the Human Research Ethics Committee of Hospital Clínico San Carlos (Madrid, Spain) and the AEMPS (Spanish Agency of Medicines and Medical Devices). The participant presented periodontal disease and required anterior sector fixed rehabilitation with dental implants. All procedures were carried out according to Good Clinical Practice requirements.

Implants were placed according to a “two-stage protocol”, in which implants are covered within the gingival flap during the osseointegration period (60 days, approximately). The surgical protocol applied for implant insertion was performed according to the implant manufacturer’s instructions. Using infiltrative local anesthesia, a full-thickness flap was elevated to perform progressive drilling with gradual enlargement of the bur hole under profuse irrigation. All implants were placed by applying an insertion torque of 50 N/cm in an immediate implant placement protocol. The following postoperative medications were prescribed: 1 day of antibiotic prophylaxis with amoxicillin (500 mg/8 h) (Clamoxyl^®^, GlaxoSmithKline S.A), analgesia with dexketoprofen (25 mg/8 h) (Enantyum^®^, Laboratorios Menarini S.A) and metamizole (575 mg) (Nolotil^®^,Boehringer Ingelheim Spaim, S.A), omeprazole (20 mg/24 h) (Teva Pharma S.L.U.) and prednisone (50 mg/24 h) (Prednisone Alonga; Sanofi-Aventis S.A.).

After an eight-week period, “secondary implant stability” or osseointegration was assessed, and implants were considered ready for functional loading. During the second stage of surgery, the coated ceramic composite abutments were placed on the titanium implants using Ti 6Al-4V ELI screws and applying a torque of 32 N/cm. Once placed, the abutments were not removed from the implants, to prevent disturbance of soft tissue healing and biologic width formation.

Following the center’s standard protocol, hygiene rules and instructions were explained in detail, as well as provided in writing.

The implants were prosthetically rehabilitated with fixed prostheses. The prostheses were individually and directly screwed to each abutment’s multi-unit connection using Ti 6Al-4V ELI screws, applying a torque of 20 N/cm. Prosthesis manufacture was performed at a professional dental prosthetics laboratory, integrally in zirconia, via CAD/CAM, after performing a standard impression procedure using customized PEEK impression copings and AISI 316 stainless steel implant analogs.

This study encompassed a 1-year follow-up period after prosthesis deliverance. During this period, milestone checks were performed to assess primary and secondary outcomes, following the protocols reported in previous work [28]. Once per week, a clinical examination was performed to assess the soft tissue inflammation. Visual signs of gingival inflammation such as redness and swelling were evaluated.

## 3. Results and Discussion

**Figure 4:**
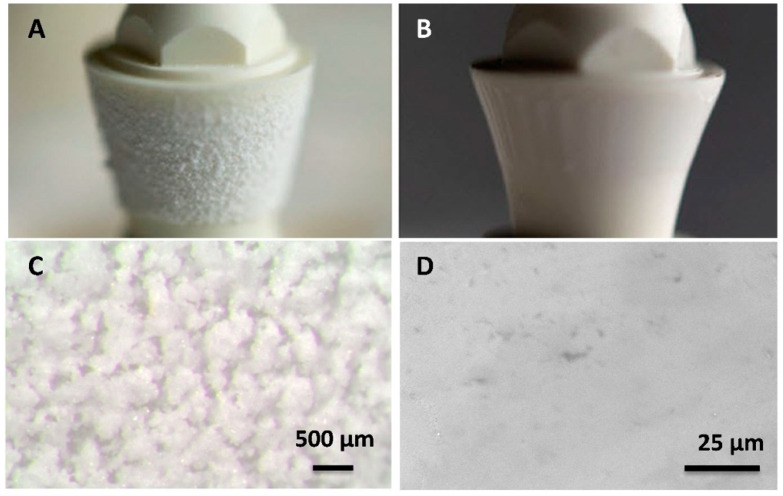
Coated abutments before (left) and after (right) thermal treatment. (**A**,**B**): General views. (**C**,**D**): Close-ups of the surfaces.

Glassy coatings were successfully obtained via the applied airbrush spraying method. The glossy finish of the coatings was clearly visible after the thermal treatment of the abutments. Figure 4 shows some images of the coated abutments, before and after thermal treatment. The total amount of glass deposited per coating was around 3 mg, determined by calculating the difference in the weight of the specimens before coating and after thermal treatment (after coating). It was possible to control the thickness of the coating by adjusting the spray pressure and the total spraying time. In preliminary experiments, we determined the optimal conditions for producing the coatings. The thickness of the coating does not affect the bactericidal properties or the cell response, but it does affect the mechanical properties. We observed delamination in coatings with a thickness greater than 80 microns. Taking into account the fact that during cooling at high temperatures (1200 °C–700 °C) the glassy coating possesses high plasticity, the difference between the expansion coefficients of the glass and the nanocomposite (12 × 10^−6^ K^−1^ ) [51] can be considered negligible, justifying the observed mechanical stability of the final coating with a thickness below 80 µm. A similar behavior was observed in the case of a Y–TZP substrate [28].

**Figure 5:**
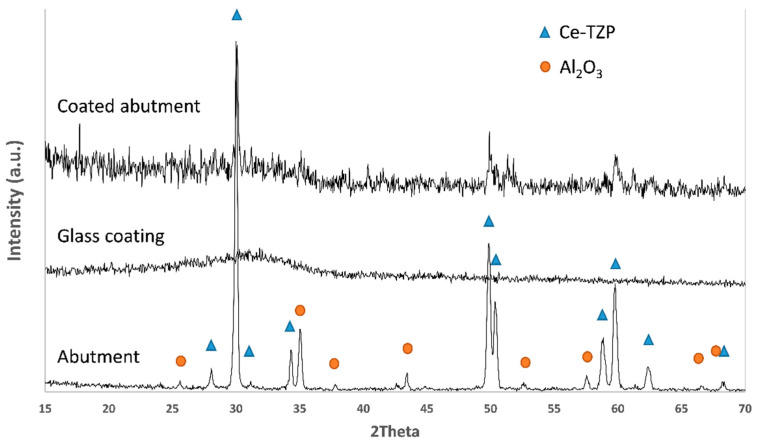
XRD pattern of the abutment, glass coating and glass-coated abutment.

Characterization by XRD (Figure 5) revealed no crystallization of the glass, which supports the experimental conditions used. The peaks that can be seen in the XRD pattern are attributed to the ceramic composite substrate. 

**Figure 6:**
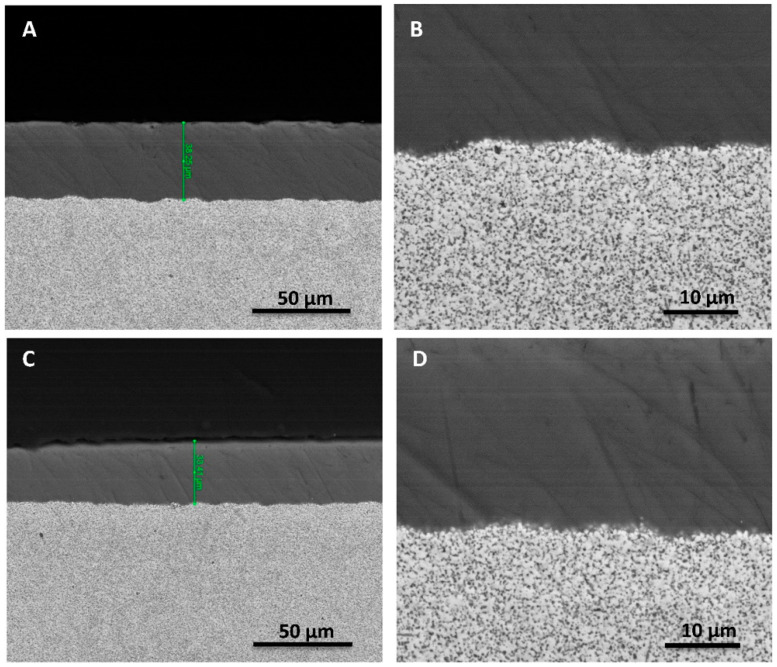
FESEM micrographs at different magnifications, showing the coating–substrate cross section. (**A**,**B**): Before the fatigue test. (**C**,**D**): After mechanical testing. No cracking is visible.

Continuous glass coatings with relatively homogeneous thicknesses (~35 µm) along the cross section were obtained without delamination or cracks at the interface with the ceramic composite substrate (Figure 6A,B).

Cytotoxicity can be considered as inversely proportional to cell viability. Cell viability was spectroscopically evaluated by quantifying the conversion of tetrazolium salts by mitochondrial dehydrogenases after incubation within extracts of coated and uncoated abutments. The cell viability was higher than 70% for both coated and uncoated abutments which means that they are not cytotoxic according to the ISO 10993-5 standard [39].

The mechanism of the antibacterial activity of the glass has been studied in previously [32,52]. This phenomenon can be attributed to the high punctual concentrations of calcium ions at the glass–membrane interface that can distort the membrane electrochemical potential gradient, thus avoiding nutrient exchange and consequently inducing the death of the cell. Moreover, in a recent study [33], we evaluated the ability of the glass and its lixiviate to show antiviral activity. Lixiviates were obtained by placing the cell culture in contact with glass powder for 24 h. After this time, the glass particles were removed by centrifugation. Then, the obtained supernatants were exposed to a fixed amount of vesicular stomatitis virus (VSV-FFP) (10^6^ infective particles). From the results obtained in this study, we can conclude that the antiviral properties cannot be explained exclusively by the substances present in the lixiviate of the glass particles. Additional virus inhibition could be explained by direct virus–material contact.

The present mechanical in vitro investigation performed on the Ce–TZP/Al_2_O_3_ abutments (compatible with commercially available Ti implants) coated with an antimicrobial glass showed the mechanical integrity of the coating after cyclic loading. The maximum load (400 N) reached during fatigue testing exceeded the range of occlusal forces documented in the premolar region of humans while chewing and swallowing under normal conditions (148 to 354 N) [53,54]. At this high cyclic load, the bonding of the coating to the substrate was found to be good in all cases after the fatigue tests, as evidenced by the lack of spalling in all samples (Figure 6C,D).

Consequently, the ceramic implant components evaluated proved their ability to survive the maximum loading forces that occur in vivo over the long term, including a sufficient “worst-case” buffer, and the antimicrobial glass-based coating was proved to have sufficient mechanical reliability and stability to survive mastication in the oral cavity over the long term.

**Figure 7:**
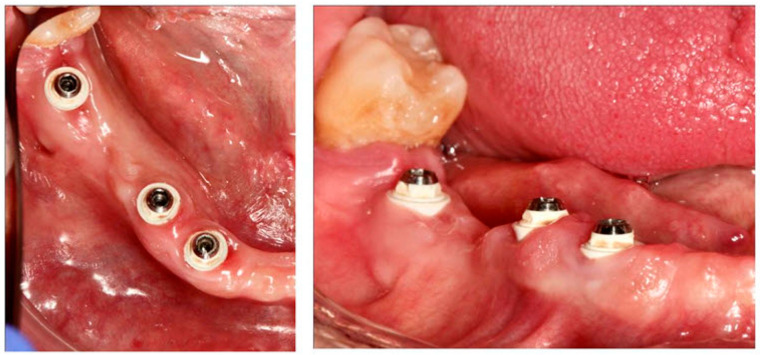
Peri-implant tissue surrounding the coated ceramic abutments one year after implant insertion.

**Figure 8:**
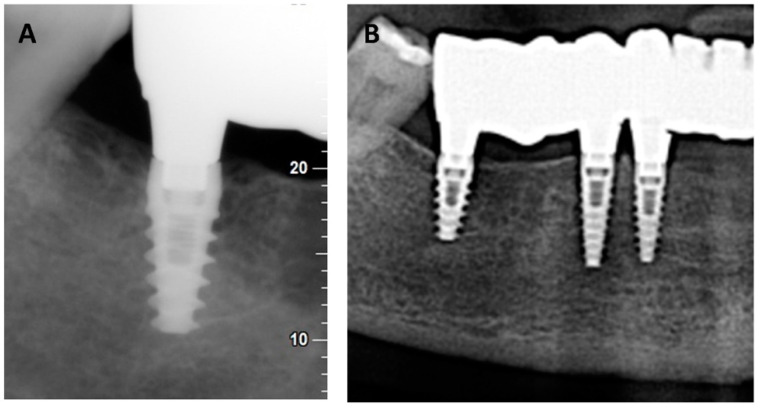
Periapical X-ray of a titanium implant with a ceramic composite abutment (**A**) and detail of the panoramic view (**B**).

The pilot clinical case showed no signs of inflammation of the peri-implant tissue surrounding the coated ceramic abutments one year after implant insertion (Figure 7). During the postoperative period, no adverse or unanticipated events occurred. No alterations were detected during the intraoral examination of the operated area. Moreover, the correct osseointegration of the implants was verified radiologically (Figure 8). Therefore, these new, biocompatible and antibacterial glassy coatings can be located on Ce–TZP/Al_2_O_3_ ceramic abutments to prevent bacterial biofilm adhesion to or in the peri-implant groove. The coating respects gingiva–abutment adhesion and prevents bacteria reaching the osseointegration area of the implant.

The transition from laboratory trials to a clinical scenario and the possible impact on peri-implantitis treatment must be considered here. However, based on the in vitro mechanical results and the preliminary clinical study, it can be assumed that the antimicrobial glass-based coating analyzed could withstand the typical physiologic masticatory forces of the anterior dentition. Moreover, the coated ceramic composite was shown to perfectly integrate and be biologically compatible with the gingiva (no gingival inflammation) in a patient prone to plaque generation.

In conclusion, coating these ceramic implant components with this antimicrobial glass seems to be a promising surface modification technique that may enable successful oral surgery, prevent infectious diseases and avoid the use of antibiotics.

## 4. Conclusions

- In the present study, mechanically stable thin antimicrobial soda–lime coatings were successfully deposited on Ce–TZP/Al_2_O_3_ abutments (compatible with commercially available Ti implants) by means of suspension spraying and subsequent thermal treatment.
- The antimicrobial coatings survived a load as high as 400 N under cyclic loading conditions over a long period of time (10 × 10^6^ cycles, equivalent to ≈20 years in vivo).
- The coating showed high biocompatibility and mechanical stability one year after implant insertion in a patient with periodontal problems.
- Therefore, these new, biocompatible and antimicrobial glassy coatings placed on ceramic composite dental implant components could be promising candidates to reduce the risk of peri-implant infections in the anterior dentition.

## Data Availability

The raw/processed data required to reproduce these findings cannot be shared at this time as the data also form part of an ongoing study.
